# Investigating the Relationship Between Eye Movement and Brain Wave Activity Using Video Games: Pilot Study

**DOI:** 10.2196/games.8908

**Published:** 2018-09-13

**Authors:** Chaoguang Wang, Gino Yu

**Affiliations:** 1 Digital Entertainment Lab School of Design Hong Kong Polytechnic University Kowloon China (Hong Kong)

**Keywords:** video games, games for health, eye movement desensitization reprocessing (EMDR), electroencephalography (EEG), player experience

## Abstract

**Background:**

All eye movements are related in one way or another to our mental processes with lateral eye movements being associated with the different hemispheres of the brain. Eye movement techniques form the basis of eye movement desensitization and reprocessing therapy, wherein forced eye movements activate neurological pathways to treat the subject.

**Objective:**

The objective of our study was to examine the relationship between players' eye movements and their brain wave activities using a video game.

**Methods:**

We used similar eye movement techniques in the form of a video game called *Lifeguard* that could potentially stimulate different eye movement mode and create a more engaging experience for the user. By designing an experiment, we further explored the differences in electroencephalogram spectral power activity for the alpha, beta, theta, delta, and gamma frequency bands in *Lifeguard* and *Tetris*.

**Results:**

The game based on eye movement technologies resulted in decreased delta power and increased beta power, but significant difference between 2 games was not found.

**Conclusions:**

The applied uses of this research could mean that eye movement desensitization and reprocessing can be conducted in a more fun and engaging way through the use of gaming technology.

## Introduction

### Background

Video games have been creating engaging experiences for entertainment purposes since 1970s. As computing power has continued to advance over the years, the sound, graphics, and complexity of video games have improved to create more stimulating experiences, and the application of games has expanded beyond entertainment to areas, such as education and health.

The use of biofeedback plays a key role in the form of video game treatment because it provides a quantitative measure of physiological state. Moreover, the use of real-time biofeedback provides a method that monitors changes in the effect of an individual during game play. By analyzing these changes and using the analysis results to influence the game, it is possible to regulate a person’s physiological metrics toward a specific state or to modify their behavior [1].

In this study, we aimed to use video game technologies, including motion graphics and emotionally charged scenarios, to explore the relationship between eye movements as well as game play and brain wave activities. Eye movement is involved in memory recall and disruption of normal eye movement patterns that form the basis of eye movement desensitization and reprocessing (EMDR) therapy, wherein forced eye movements activate neurological pathways to treat the participant [2-5]. Designing similar eye movement activities into video game play could create a more engaging experience for the user.

### Games Designed to Improve Health

The experiences created by video games activate mental processes that regulate the autonomic nervous and endocrine systems; for example, in-game situations that induce fear activate the sympathetic nervous system and the endogenous production of adrenaline, cortisol, and norepinephrine.

Thus, video games provide a possible alternative to prescribing pharmaceuticals for treating behavioral and neuropsychiatric disorders. By entraining somatic awareness and behavioral skills acquired via game experience, individuals can slightly moderate their behavior in real-life situations and create a positive lifestyle change. Video games have been used in a beneficial manner to help treat overweight-related behaviors [6], prevention of alcoholism in adolescents [7], and posttraumatic stress disorder (PTSD) [8,9].

By specifically designed games to immerse a participant in a virtual reality environment, the PTSD symptoms of war veterans can be significantly reduced [8]. Instead of placing them in a potentially harmful situation, this will highlight the underlying negative cognitive patterns in a safe way. It has also been demonstrated that playing the video game *Tetris* for a short period of time immediately after an emotional experience can reduce its psychological impact [10]. In this experiment, after viewing traumatic materials, the unwanted, involuntary memory flashbacks of the participants who played *Tetris* for 10 minutes reduced.

Playing video games that promote learning sequences have reduced attention deficit hyperactivity disorder (ADHD) symptoms. Weerdmeester et al have found that 73 children with elevated ADHD symptoms improved in several areas with only a short amount of gameplay (1.5 hours) compared with those who played a game without ADHD-focused training components [11]. Peijnenborgh et al have developed a computer game called *Timo’s Adventure* as an assessment tool for cognitive functions and tested its validity in normal developing children and children with ADHD [12]. The result has shown that the game could be a valid tool in assessing specific strengths and weaknesses of young children with ADHD. Meanwhile, Bul et al [13] have designed and implemented a serious game called *Plan-It Commander* as an adjunct to treatment for children with ADHD. They have found that several skills of the participants who received a serious game intervention significantly improved.

Previous studies have shown that PTSD and ADHD are correlated to brain activity. Veterans with PTSD are more likely to have decreased alpha power and increased beta power [14]. In individuals with ADHD, the aim is to decrease the theta band and increase the beta band, which corresponds to an alert and focused but relaxed state [15,16]. The horizontal eye movement training can also increase alpha amplitude and decrease delta amplitude, correlating with the subjective improvement of sleep quality and well-being as well as a sense of optimism [17].

### Eye Movement

To explore the relationship between eye movements as well as game play and brain wave characteristics, the basic concepts of eye movement must first be understood. Mental processes stimulate all eye movements. Neurological activities, such as memory access, correlate to specific eye movement patterns.

Dilts et al has used electrodes to track both the eye movements and brain wave activities of participants who were asked to answer a series of questions about sight, sound, or kinesthetic feelings [2]. Specific eye movements were found to be correlated with brain activity during different cognitive tasks with shifts in eye movements directly relating to the part of the brain they were using; for example, lateral eye movements to either side are an indicator of internal auditory activity (ie, remembered sounds and words). Eye movements down and to the left are found to indicate internal dialogue or inner self-talk. Upward eye movements to either side are believed to indicate internal visual activity (ie, constructed imagery and visual fantasy). Moreover, Dilts et al has pointed out that for left-handed individuals, the down left and are merely reversed in this model.

EMDR is a form of psychotherapy that has been designed to relieve the symptoms of traumatic events, such as accidents, experiences in military combat, or rape. Shapiro has found that distressing or disturbing thoughts could be eliminated with the pairing of diagonal upward to and fro eye movements [3]. In a study involving 70 individuals, a procedure that involved the use of various eye movements and the patients’ distressing thoughts and images were used to unblock stalled emotional processing. Despite the fact that it is a relatively new treatment, studies have shown that the effectiveness of EMDR is similar to that of the more traditional trauma-focused cognitive behavioral therapy (CBT) in the treatment of PTSD in adults [4]. In addition, EMDR therapy has helped manage previously untreatable cases of PTSD [5].

EMDR is a therapy that influences emotional processing via eye movement, and using the basic principles of game design will influence the user’s emotions and memories. Electroencephalogram (EEG) measures the underlying physiological responses to eye movement therapy. Biofeedback can be used to stimulate eye movements toward specific brain states and explore its implications on memory, emotions, and engagement.

### Electroencephalography Technology

An EEG reading measures the action potentials occurring on the cortical surface of the brain due to neural activation, thus providing brain activity measurement. Temporal and spatial activities correlate to specific mental states, as listed below:

1. *Delta (1-4 Hz)*: Deep sleep and unconscious processes, such as fatigue or trance
2. *Theta (4-8 Hz)*: Daydreaming, creativity, intuition, memory recall, emotions, and sensations
3. *Alpha (8-14 Hz)*: Cortical inactivity and mental idleness as well as demand of attention
4. *Beta (14-30 Hz)*: Frontal cortex and cognitive processes, decision making, problem solving, and information processing

EEG studies have found increased frontal and parietal alpha power activities during a racing game [18] and elevated theta activity during long gaming tasks [19]. Nacke et al compared 3 different level design conditions (boredom, immersion, and flow) by measuring the patterns of EEG spectral power [20]. The result has indicated that the immersion-level design elicited more activity in the theta band. Rani et al analyzed 3 levels of intensity for different emotions based on the EEG data obtained using the *Pong* game and anagram puzzles [21]. Sourina et al used EEG signals to continuously assess the emotional state of players and developed the game for stress management called *Pipe* [22]. Using EEG signals, Chanel et al assessed 3 different emotions (boredom, engagement, and anxiety) corresponding to 3 difficulty levels of *Tetris,* and the accuracy increased up to 63% [23]. Ravaja et al showed that different EEG data are triggered by wounding and killing events in a digital console game; for example, wounding resulted in an increase in occipital theta activity [24].

These results motivate us to test the following 2 hypotheses in this study: H1, the beta power will increase with playing the EMDR game and H2, the theta power will decrease with playing the EMDR game.

## Methods

### Game Design and Development

**Figure 1:**
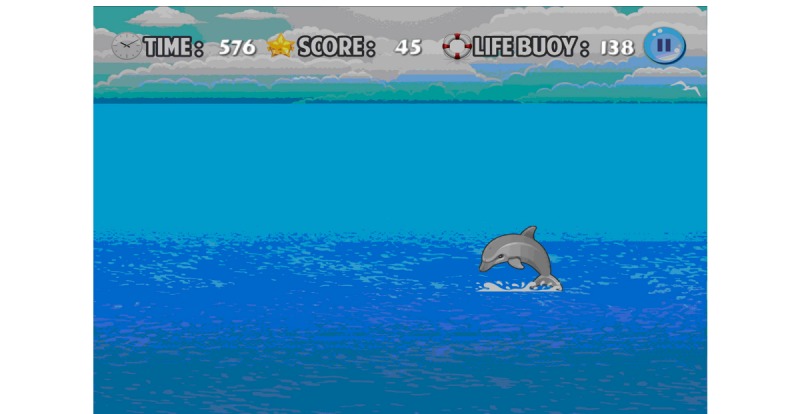
Screenshot of *Lifeguard*.

We designed a video game called *Lifeguard* to induce eye movements, as shown in Figure 1, to create more engaging experience among eye movement processes. During the game play, the players need to stare at the floating targets, which will provoke 4 different eye movement modes, including *right-left, up-down, up right-up left, and down right-down left*.

A player rescues the floating target by clicking on it, and each click will decrease a life buoy number. They must decide if it is the right time to throw the life buoy out to rescue the floating, whereas the rescuing of humans and animals will bring reward points. However, other things, such as fish, will just waste the limited number of life buoys. If they click on other places in the screen, the life buoy will be wasted as well. Different targets will bring different numbers of reward. Some particular targets will bring extremely high reward points, such as a baby and old man, and this randomization will increase the fun of surprise and variation for the whole process of game play. There is also a time limit of 10 minutes for each game session. The game will be over when the number of life buoys becomes zero or the player runs out of time. In addition, the goal of the game is to get maximum possible reward points via rescuing targets within the limit of both life buoy number and time. A summary will show the record of the players in this game session, including reward points and the total number of targets rescued, which can be compared across different sessions or different players.

**Figure 2:**
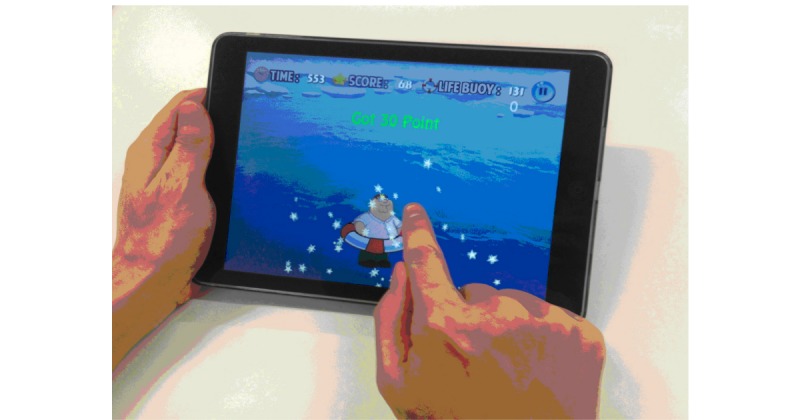
Playing *Lifeguard* on an iPad.

We finished the design document of *Lifeguard* and collaborated with a game company to do the art and coding work for this game. Meanwhile, we oversaw the whole process. A playable iOS version of *Lifeguard* has been launched, and it can be run on the platform of iPad or iPhone, as shown in Figure 2.

### Electroencephalogram Measurement of the Eye Movement Game

To better understand the physiological mechanism behind eye movement, real-time biometric measurement is used to monitor the participants while playing the video game. We recorded brain activity using 64 electrodes during the duration of game play, allowing us to track what eye movements stimulate the brain and what kind of brainwaves are being produced.

In particular, we wanted to the compare eye movement game with *Tetris* because a previous study has shown that Tetris can be used for the treatment of PTSD [10]. The experiment was designed to examine if the EMDR-based game will stimulate different measurable brain wave patterns compared with the commercial game *Tetris*. We mapped out the differences in EEG spectral power activity for the alpha, beta, theta, delta, and gamma frequency bands evoked by the 2 games. The version of *Tetris* used herein is from the Apple Store [25].

### Experimental Design

We used a repeated-measures within-subject design with game playing as an independent variable in 2 conditions (*Lifeguard* and *Tetris*). Each participant played 2 games in a random order (a sequence of AB or BA) to eliminate sequence effects. The dependent variables included the collected and estimated EEG spectral power.

**Figure 3:**
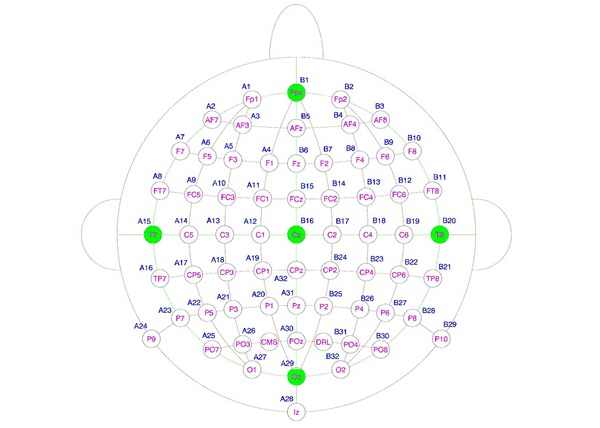
Position of 64 electrodes.

With the use of electrodes attached on a player’s scalp, EEG will determine the electrical impulses generated by the brain during a given sequence. EEG readings obtained real-time workable measures of engagement and emotion corresponding to different sequences represented in the game. Typically, an EEG measures the voltage recorded between the electrodes placed in standard position on the scalp. As shown in Figure 3, we measured brain activity using 64 scalp pin-type active electrodes and with common mode sense active electrode and driven right leg passive electrode that is equivalent to ground electrodes. Each electrode is letter coded to indicate its position when distributed over the head, such as frontal, parietal, temporal, occipital, or central. Electrooculogram and video-oculography are recorded to correct artifacts from eye movements by placing flat-type active electrodes on the lift and above and below the lift eye. The raw EEG signal is captured with the ActiView acquisition software.

### Participants

Overall, 11 healthy participants were recruited from the Hong Kong Polytechnic University, and they completed the experiment in the EEG laboratory in the School of Design of Hong Kong Polytechnic University. The participants included 7 women and 4 men aged 24-30 years. All 11 participants are right-handed, and they have experience in playing video games.

### Procedure

First, the participants were asked to fill out a pre-experiment questionnaire about their demographic characteristics, such as gender and age, and game experience. Before the experiment, the instructor provided a brief introduction about the experiment and EEG measurement. The participants were then asked to seat in a comfortable sofa, and the electrodes were then attached. The participants were asked to relax for approximately 3 minutes, during which the baseline recordings were obtained. Then, the participants played 2 game sessions each for approximately 7-10 minutes (maximum time) on an iPad with the EEG measurement.

## Results

Raw EEG signals were processed using the BESA software (Besa GmbH, Gräfelfing, Germany). The average power estimates were calculated using fast Fourier transformation with the following frequency bands: delta, 1-4 Hz; theta, 4-8 Hz; alpha, 8-14 Hz; beta, 10-30 Hz; and gamma, 30-50 Hz. The spectral power estimates were then averaged for all 64 electrodes for each frequency band.

**Figure 4:**
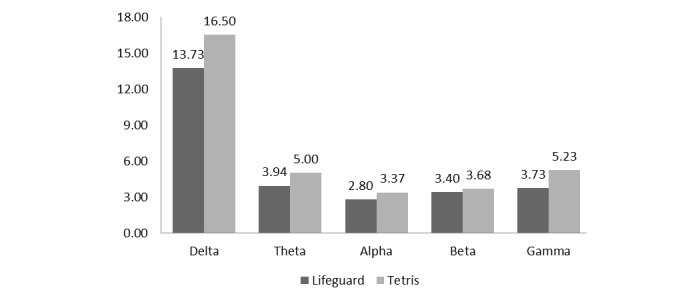
Electroencephalogram frequency of *Lifeguard* and *Tetris* playing.

A series of *t* test was performed to examine the differences across all 5 band power averages with 2 games as independent variables and different frequency bands as dependent variables. As shown in Figure 4, the eye movement game decreased the delta frequency bands, whereas the score of alpha bands was extremely similar. However, as indicated in Table 1, no statistical significance was revealed.

**Table 1:** *t* test of electroencephalogram frequency.

Frequency and game		Mean^a^	*t* test	*P* value
**Delta**			0.60	.57
	*Lifeguard*	13.73		
	*Tetris*	16.50		
**Theta**			1.06	.32
	*Lifeguard*	3.94		
	*Tetris*	5.00		
**Alpha**			0.91	.39
	*Lifeguard*	2.80		
	*Tetris*	3.37		
**Beta**			0.29	.78
	*Lifeguard*	3.40		
	*Tetris*	3.68		
**Gamma**			−0.86	.41
	*Lifeguard*	3.73		
	*Tetris*	5.23		

^a^Mean: all the values indicate average power calculated.

## Discussion

We designed and implemented *Lifeguard* based on eye movements, and the EEG results of the players of this game were compared with those of the players of *Tetris*. However, we did not find a significant difference in the EEG frequency stimulated by playing *Lifeguard* and *Tetris*. Our results did not support H1 and H2. However, we have observed some trends that could be used as a basis for future research.

When interpreting biometric data collected from game playing, it is important to understand the relationship between mental processes and body responses. The players’ body is still present in the real world while playing a video game; therefore, it responds to specific in-game elements and external activities, anticipation, or something not otherwise observed. Thus, the physiological response to video games is a many-to-one relationship, wherein one body response may be associated with several mental effects or processes [26]; for example, when recording the biometric data of a player, room temperature, movement, drugs, and noise may influence brain waves and bring contextual bias in the interpretation of the result. Without a higher degree of experimental control, it will be difficult for researchers to make assumptions about the players’ mental processes based on their body responses [27].

This study only used *Tetris*, which is a visual space game. For future research, more video games, such as action, puzzle, and shooting games, may be compared with *Lifeguard* to explore their difference. In addition, further studies should be conducted to compare the EEG features of different eye movement modes stimulated by game playing, such as horizontal moving and vertical moving.

The use of games in exploring the relationship between eye movement and brain wave activities can also lead to improved eye movement techniques for existing treatments, such as EMDR, or new techniques to treat other ailments or disabilities.

## Abbreviations

ADHD: attention deficit hyperactivity disorder
CBT: cognitive behavioral therapy
EEG: electroencephalogram
EMDR: eye movement desensitization and reprocessing
PTSD: posttraumatic stress disorder

## References

[1] Yu G, Martin JA, Chai P (2012). Shifting worldview using video game technologies. Video Game Play and Consciousness.

[2] Dilts R, Grinder J, Bandler R, DeLozier J (1980). Neuro-Linguistic Programming Volume 1: The Study of the Structure of Subjective Experience.

[3] Shapiro F (2001). Eye Movement Desensitisation and Reprocessing: Basic Principles, Protocols and Procedures.

[4] Seidler GH, Wagner FE (2006). Comparing the efficacy of EMDR and trauma-focused cognitive-behavioral therapy in the treatment of PTSD: a meta-analytic study. Psychol Med.

[5] MacCulloch M (1999). Eye movement desensitisation and reprocessing. Advances in Psychiatric Treatment.

[6] Spook J, Paulussen T, Kok G, van EP (2016). Evaluation of a Serious Self-Regulation Game Intervention for Overweight-Related Behaviors (“Balance It”): A Pilot Study. J Med Internet Res.

[7] Drost RMWA, Paulus ATG, Jander AF, Mercken L, de VH, Ruwaard D, Evers SMAA (2016). A Web-Based Computer-Tailored Alcohol Prevention Program for Adolescents: Cost-Effectiveness and Intersectoral Costs and Benefits. J Med Internet Res.

[8] Rothbaum B O, Hodges L, Alarcon R, Ready D, Shahar F, Graap K, Pair J, Hebert P, Gotz D, Wills B, Baltzell D (1999). Virtual reality exposure therapy for PTSD Vietnam Veterans: a case study. J Trauma Stress.

[9] Reger G, Gahm G (2008). Virtual reality exposure therapy for active duty soldiers. J Clin Psychol.

[10] Holmes EA, James EL, Coode-Bate T, Deeprose C (2009). Can playing the computer game “Tetris” reduce the build-up of flashbacks for trauma? A proposal from cognitive science. PLoS One.

[11] Weerdmeester Joanneke, Cima Maaike, Granic Isabela, Hashemian Yasaman, Gotsis Marientina (2016). A Feasibility Study on the Effectiveness of a Full-Body Videogame Intervention for Decreasing Attention Deficit Hyperactivity Disorder Symptoms. Games Health J.

[12] Peijnenborgh JC, Hurks PP, Aldenkamp AP, van DSED, Rauterberg G, Vles JS, Hendriksen JG (2016). A Study on the Validity of a Computer-Based Game to Assess Cognitive Processes, Reward Mechanisms, and Time Perception in Children Aged 4-8 Years. JMIR Serious Games.

[13] Bul KCM, Kato PM, Van DOS, Danckaerts M, Vreeke LJ, Willems A, van OHJJ, Van DHR, Birnie D, Van ATAMJ, Franken IHA, Maras A (2016). Behavioral Outcome Effects of Serious Gaming as an Adjunct to Treatment for Children With Attention-Deficit/Hyperactivity Disorder: A Randomized Controlled Trial. J Med Internet Res.

[14] Jokić-Begić N, Begić D (2003). Quantitative electroencephalogram (qEEG) in combat veterans with post-traumatic stress disorder (PTSD). Nord J Psychiatry.

[15] Lubar J F, Swartwood M O, Swartwood J N, O'Donnell P H (1995). Evaluation of the effectiveness of EEG neurofeedback training for ADHD in a clinical setting as measured by changes in T.O.V.A. scores, behavioral ratings, and WISC-R performance. Biofeedback Self Regul.

[16] Gevensleben H, Holl B, Albrecht B, Vogel C, Schlamp D, Kratz O, Studer P, Rothenberger A, Moll GH, Heinrich H (2009). Is neurofeedback an efficacious treatment for ADHD? A randomised controlled clinical trial. J Child Psychol Psychiatry.

[17] Choi KM, Min JA, Park GH, Lee SH, Chae JH (2011). The effects of horizontal eye movement on mental health indices and psychophysiological activities in healthy subjects. Korean J Biol Psychiatry.

[18] Schier MA (2000). Changes in EEG alpha power during simulated driving: a demonstration. Int J Psychophysiol.

[19] Sheikholeslami C, Yuan H, He EJ, Bai X, Yang L, He B (2007). A high resolution EEG study of dynamic brain activity during video game play. Proceedings of 29th Annual International Conference IEEE Engineering in Medicine and Biology Society.

[20] Nacke LE, Stellmach S, Lindley CA (2010). Electroencephalographic assessment of player experience: a pilot study in affective ludology. Simulation & Gaming.

[21] Rani P, Sarkar N, Liu C (2005). Maintaining optimal challenge in computer games through real-time physiological feedback. Proceedings of the 11th International Conference on Human Computer Interaction.

[22] Sourina O, Liu Y, Wang Q, Nguyen M (2011). EEG-based personalized digital experience. Universal access in human-computer interaction. Users diversity. Lecture notes in computer science.

[23] Chanel G, Rebetez C, Bétrancourt M, Pun T (2011). Emotion Assessment From Physiological Signals for Adaptation of Game Difficulty. IEEE Transactions on Systems, Man, and Cybernetics-Part A: Systems and Humans.

[24] Ravaja N, Turpeinen M, Saari T, Puttonen S, Keltikangas-Järvinen L (2008). The psychophysiology of James Bond: phasic emotional responses to violent video game events. Emotion.

[25] Apple Inc.

[26] Cacioppo J, Tassinary L, Berntson G (2007). Handbook of Psychophysiology, 3rd edition.

[27] Gow J, Cairns P, Colton S, Miller P, Baumgarten R (2010). Capturing player experience with post-game commentaries. In Proc. 3rd Int. Conf. on Computer Games, Multimedia & Allied Technology.

